# Ensemble-Learning Framework for Intrusion Detection to Enhance Internet of Things’ Devices Security

**DOI:** 10.3390/s23125568

**Published:** 2023-06-14

**Authors:** Yazeed Alotaibi, Mohammad Ilyas

**Affiliations:** Department of Electrical Engineering and Computer Science, Florida Atlantic University, 777 Glades Road, Boca Raton, FL 33431, USA; ilyas@fau.edu

**Keywords:** ensemble learning, machine learning, internet of things, security, intrusion detection system

## Abstract

The Internet of Things (IoT) comprises a network of interconnected nodes constantly communicating, exchanging, and transferring data over various network protocols. Studies have shown that these protocols pose a severe threat (Cyber-attacks) to the security of data transmitted due to their ease of exploitation. In this research, we aim to contribute to the literature by improving the Intrusion Detection System (IDS) detection efficiency. In order to improve the efficiency of the IDS, a binary classification of normal and abnormal IoT traffic is constructed to enhance the IDS performance. Our method employs various supervised ML algorithms and ensemble classifiers. The proposed model was trained on TON-IoT network traffic datasets. Four of the trained ML-supervised models have achieved the highest accurate outcomes; Random Forest, Decision Tree, Logistic Regression, and K-Nearest Neighbor. These four classifiers are fed to two ensemble approaches: voting and stacking. The ensemble approaches were evaluated using the evaluation metrics and compared for their efficacy on this classification problem. The accuracy of the ensemble classifiers was higher than that of the individual models. This improvement can be attributed to ensemble learning strategies that leverage diverse learning mechanisms with varying capabilities. By combining these strategies, we were able to enhance the reliability of our predictions while reducing the occurrence of classification errors. The experimental results show that the framework can improve the efficiency of the Intrusion Detection System, achieving an accuracy rate of 0.9863.

## 1. Introduction

The Internet of Things (IoT) has grown exponentially due to technology’s evolution. The IoT facilitates people’s lives by providing and enhancing connectivity that supports the automation aspect of several human services. Millions of interconnected devices use the IoT to communicate, transfer, share, collect, and analyze data from several domains [1]. While involving the internet as the main factor in the technology’s fields, it opens a new platform for cybercriminals [2]. Therefore, enhancing security and employing artificial intelligence innovations and technologies led to a protected and reliable IoT infrastructure.

In addition, the IoT architecture contains three main layers: the application, network, and user experience levels. The Perception layer has responsibility for every task, from utilizing the sensors to gathering the information, but it is also susceptible to multiple attacks due to its central role. Physical attacks on sensor-equipped equipment, unauthorized entry into the infrastructure, and other forms of physical attack are prevalent. The Network layer allows the devices fitted with sensors to communicate and exchange data with the gateways and other IoT devices via wireless technologies such as Wi-Fi, 3G, and 4G. The most common attacks faced by the Network layer are distributed denial-of-service (DDoS), denial-of-service (DOS), Man of the Middle, information theft, and gateways attacks [1,3].

Despite IoT’s high dependence on the Internet as its primary communication network, the Internet of Things faces a new generation of innovative cybercriminals [4]. The IoT consists of a network of interconnected nodes that constantly communicate with one another and process information over a variety of network protocols. Because of the ease with which these protocols can be exploited, it has been discovered that they pose a considerable risk to the confidentiality of the transferred data [5].

Researchers have developed and invented new security technologies based on AI technologies, such as ML, to detect and prevent such attacks. Unlike traditional firewalls and detection techniques, ML can handle big data environments [6,7]. Moreover, the ML is a well-known solution to address detection attacks and classification issues. ML might be considered the most suitable solution to secure and stabilize IoT network traffic [8]. ML has several techniques, such as regression, and classification [9]. In addition to preventing and detecting novel attacks, ML provides an appropriate strategy for securing IoT networks with its collection of supervised, unsupervised, and reinforcement techniques and algorithms [10].

One of the recent machine learning techniques is the prevention technique, such as the Intrusion detection system (IDS).The primary function of the IDS is to identify and report the normal or abnormal behavior of the traffic messages [1,11]. In addition, the IoT object is vulnerable to attacks because they rely on wireless communication protocols [12]. Attacks on the IoT affect all IoT network components, as opposed to the typical local network, where attacks affect specific nodes [13].

Furthermore, it is still unclear which machine learning techniques are more reliable and efficient for building a dynamic IDS. IoT IDS have been the focus of extensive study and research, with numerous machine learning and deep learning approaches being applied to a variety of datasets to determine their efficacy [14]. Time is a critical component of an effective IoT attack prevention system, so it’s essential to consider ways to speed up the process of building and training the system. This can be completed by decreasing the computational time required by intrusion detection systems [11].

While the ML model scenarios do not show accurate and satisfactory results, the ensemble methods were employed to address this issue since it is important to combine several methods to avoid the instability aspect [15]. The primary objective of the ensemble is to enhance efficiency by combining the classification of several ML base classifiers [16]. When the performance of the ML classifiers is considered weak or low, the ensemble method is applied to combine the weak classifiers to construct a robust predictive model and enhance the performance [17]. Ensemble learning has been applied to several datasets, and it is communally used to construct intrusion detection approaches [17].

In addition, the ML detection system’s capability depends on the quality of the database, so collecting or generating a credible dataset from the IoT communications environment at various levels that involves realistic attacks and regular traffic is a necessary step. Recently, a group of datasets, TON-IoT [18,19,20,21,22,23,24,25], which were collected from multiple IoT devices, have been tested and authenticated in a cyber lab to be more reliable to be applied to ML approaches.

In this study, we propose an IDS built with algorithms that use machine learning specifically for detecting intrusion attacks in IoT network traffic. Our approach’s main goal is to enhance the accuracy of attack detection. We constructed and evaluated four supervised machine learning models to classify IoT network traffic as normal or abnormal. In addition, we used two ensemble techniques, voting and stacking, to merge these supervised models. Through the use of ensemble learning, we facilitate collaboration and improve classification performance between learning mechanisms with distinct capabilities. Effectiveness in classification tasks is enhanced by the ensemble learning method, which encourages the cooperation and reinforcement of several learning systems. Therefore, the paper’s contributions are as follows:

1. Proposing a binary classification of IoT device network traffic as normal or abnormal.
2. Applying feature selection methods to improve the IDS performance of IoT network devices. Hence, we examined multiple ML algorithms to determine the most accurate and efficient learners for building an efficient IDS to detect attacks on IoT devices within IoT network data.
3. Constructing and assessing four supervised models using data preprocessing and feature selection methods. This group of models consists of Random Forests, Decision Trees, Logistic Regression, and K-Nearest Neighbor. In addition, by combining the four supervised ML models, we employ two ensemble methods to improve the efficiency of the proposed model. The performance evaluation includes accuracy, precision, recall, and F1-score metrics.
4. The model we constructed improved the performance of the detection technique compared with the most recent study that used the same dataset.

This paper is organized as follows: Section 2 highlights previous work on machine learning-based and ensemble-based models for attack categorization, and Section 3 provides a brief background of the used techniques, methods, and several machine learning algorithms. Section 4 illustrates the methodology in detail. Section 5 covers our findings, comparisons, discussion, and evaluation method. Finally, Section 6 describes and outlines the conclusion of the entire study and the planned future work.

## 2. Related Work

The IoT transforms our daily activities by providing a mechanism for managing physical objects on the edge. IoT is one of the remarkable innovations in our era to make and support systems that are smart by improving system performance and reducing human involvement by automating several domains of services [26]. While the IoT environment includes several devices and objects, it allows these objects to communicate and be interconnected. These connected IoT objects communicate and exchange data via the internet and can be remotely accessible, which puts them at high risk of being targeted by malicious attacks and unauthorized access [27].

Enhancing the security aspect will protect the IoT architecture from attacks. Preventing these attacks requires detection techniques to expose intrusions or malicious activities within the IoT network. The IDS is an efficient technique for detecting attacks and malicious activities. The ensemble machine learning technique is considered one technique that provides an efficient and accurate solution to detecting and preventing attacks or malicious activities [20]. Recently, security detection technologies based on artificial intelligence innovations have become the primary technologies and modern techniques. Thus, artificial intelligence techniques driven by extensive data analysis can detect anomalies and malicious activities more accurately and comprehensively [6].

In the research paper [6,28,29,30], an ensemble-based model is used to detect and prevent attacks by applying several machine learning classification algorithms such as SVM, J48, and DT. Several feature selection methods are applied in proposed model to select the features that are predicted to be the most relevant, such as swarm optimization. The KDD99 dataset has been used to select the nine features that were predicted to be the most relevant. Furthermore, they proposed a model with an accuracy higher than 90%. Another study in ref. [29] implemented different algorithms, LR, Gradient boosting, and DT, and applied Gradient Boosting to the stacking classifier of ensemble learning. While the most relevant features must be selected among the CICIDS2018 dataset, Chi-square analysis was performed to zero in on these 23 most important features. As a result, the suggested model outperformed seven other classifiers, with a detection rate of 98.8% and a F-measure score of 97.1% [28,31].

Another research paper [32,33] compared seven different algorithms. The BoT-IoT dataset was used with machine learning algorithms for binary and multiclass prediction. While the proposed model produced and presented the RF as the best algorithm in binary prediction, the KNN showed that it gave the highest accuracy in multiclass prediction. The research in ref. [34] highlighted that the ensemble machine learning, neural networks, and kernel methods had been applied to detect anomalies and malicious activities in the IoT architecture. The ensemble machine learning methods also outperform them in accuracy and detection rates.

While IoT is dealing with vast amounts of data, it is required to employ AI techniques to protect and secure the IoT environment and maintain the speed of exchanging and transforming the data [35]. Moreover, several public and private sectors use the internet to exchange data. When the data is transferred unencrypted via the internet, it will cause privacy issues and lead to data being hacked. While encryption is an essential aspect of transmitting data over the network. Thus, improving the Caesar cipher method creatively by applying an arithmetic model to switch to ciphertext has been accomplished [36,37].

Another research paper [38] highlighted that the authentication aspect is one of the most essential and significant factors in the security aspect. While the data has to be accessed by an authentic key to grant access, enhancing the authentication aspect of the image by promoting the security of the invisible watermarking data. Furthermore, research in ref. [39] has stated that data confidentiality is a critical issue in the cloud computing domain. While it depends entirely on the internet, it has several security issues. The most important issue out of them is the access control issue, which leads to data exploitation, and the time to access the data will be high.

The IoT has created a connected network of devices that involves heterogeneous objects from various aspects. While in the IoT, the network has no unified protocols or standards. Therefore, it is challenging to allow full security measures for those devices. While traditional security protocols provide optimal protection for the IoT architecture against threats on the Internet. Thus, constructing an efficient IDS relying on Deep Learning (DL) techniques and technologies is promising. Research papers [40,41] highlighted that applying novel DL techniques and frameworks, besides enhancing the existing DL models, presents remarkable results and strengthens the IDS performance. While it has an accurate and high prediction rate, it can assist in the maintenance aspect of the IoT network.

An IDS is an acronym for an intrusion detection system, which is focused on behavior detection to handle abnormal traffic. While IDS deals with the network’s behavior in terms of attacks, it is established to learn from this behavior pattern using several techniques. Thus, the system will recognize any violated network traffic that can lead to an attack from this pattern [42,43].

There are two different layers of the Botnet attack classification, which are network-based or hot-based [34,44,45]. While the authors emphasized that the hot-based type is considered less realistic, the authors relied on the network layer to propose a comprehensive IoT attack detection and classification model. The suggested model used six supervised Machine Learning methods to develop an IDS: the ensemble learning technique was in three of them, the neural network used two, and the kernel used one. While they have relied on two standard attack detection datasets to observe their proposed model, NSL-KDD and distilled-Kitsune-2018, the proposed model produced results that were considered the best by 1–20% from any other prior work.

The recent anomaly detection studies [46,47,48,49,50] highlighted that the ensemble learning model is applied to enhance the performance of the current anomaly detection techniques. While the ensemble model combines and uses multiple algorithms for efficient predictive performance, it outperforms and is better than the single learning algorithm. Furthermore, the research in [50] presented an intrusion detection model to detect attacks and anomalies by applying single and ensemble-based learning implemented on the UNSW-NB15 dataset. The model achieved 99% accuracy. Although few studies address the imbalanced data issue in IoT anomaly detection [51], the research in [49] presented an ensemble detection model to detect outliers to address the imbalanced data issue by extracting the in-depth features through a stacked autoencoder (SAE) and inserting them into a probabilistic neural network of the ensemble for single and multiple outlier detection. Thus, reliance on the SAE enhances performance and stability. Table 1 highlights and summarizes the recent research on machine learning and ensemble techniques in terms of their approach and the types of datasets that have been relied on.

The comparison process of recent machine learning models that relied on the ensemble method is necessary to emphasize that the proposed techniques and approaches are significantly improving performance and building an efficient IDS. Table 2 presents recent ML ensemble methods to discuss and highlight the recent prior work that will be used in the results section.

While Table 2 and Table 3 present a comparison process between the proposed model and the prior work. To the best of our knowledge, the proposed work’s novelty is that, besides the analysis, the comparison to prior research, the preprocessing process with its applied methods, the selected feature based on the applied feature selection methods, the applied ML supervised models (RF, DT, LR, KNN), and the comparison between the two ensemble approaches. The proposed approach produces significantly improved performance. A recall is 98.60 percent, precision is 98.2 percent, the F1 score is 98.61 percent, and accuracy is 98.63 percent. According to these measurements, advancements have been made relative to earlier studies’ findings. The suggested model is an ensemble of several different algorithms, including the Random Forest (RF), K-Nearest Neighbors (KNN), Logistic Regression (LR), and Decision Tree (DT). By combining several algorithms into an “ensemble”, the model takes advantage of their strengths to achieve better results than would be possible with a single algorithm alone. Table 3 presents the recent Ensemble-learning based methods compared with the proposed approach.

## 3. Background Studies

### 3.1. Intrusion Detection System (IDS)

An IDS is an acronym for an intrusion detection system, which is focused on behavior detection to handle anomalies or attacks [42]. While IDS deals with the network’s behavior in terms of attacks, it is established to learn from this behavior pattern using several techniques. Thus, the system will recognize any violated network traffic from this pattern that can lead to an attack on the infrastructure [42].

### 3.2. Supervised Models for IDS

While recent researchers have implemented various machine learning algorithms and techniques to build an efficient IDS, there are some challenges. It is considered risky for the research community to share network data because it may contain confidential or sensitive information. Thus, the lack of training data will impact the ML implementation to build an IDS system [42].

### 3.3. Ensemble Learning (EL)

The ensemble learning method is a way to view all the learning techniques and algorithms simultaneously rather than deploy them individually [17]. Recently, EL has been used for many predictions and forecasting applications, so it has been applied to address several complex issues. EL relies on a set of combined classifiers or predictors instead of single classifiers, so these sets of classifiers are trained and learned from the conducted patterns to address the same issue and get better results [17].

In addition, the ensemble methods that could be applied to IDS approaches are as follow:

#### 3.3.1. Voting

Is every lower-level classifier for its prediction casts a vote, so the winner is the prediction with the most votes [58].

#### 3.3.2. Stacking

A learning method is employed to combine the predictions in the voting process. The resulting meta-level classifier is then utilized to construct the final prediction from the predictions of the basis classifiers [58].

#### 3.3.3. Boosting

Starts with the original data set and uses a learning algorithm to construct a classifier. A new classifier is built using the same learning process after increasing the weights of the mistakenly classified tasks. The method is repeated several times. The classifiers are then combined using weighted voting [58].

In the methodology section, we explained the ensemble learning approaches that applied to our proposed model with the selected ensemble methods.

## 4. Methodology

### 4.1. Experimental Environment

The framework was implemented using Jupyter Notebook as the development environment. Within the Anaconda environment, Jupyter Notebook is a widely used tool. Python was used to create the actual code for the scheme. Python was selected because of how effectively it works, how well it scales, and how stable it is. In addition, Python offers a variety of useful metrics for evaluation, which were utilized here effectively.

While the proposed model includes several methods and algorithms, the study’s dataset, feature selection techniques, classification algorithms, ensemble approach, and the selected ensemble methods are all detailed here.

We propose a binary classification of network traffic from the Internet of Things devices as normal or abnormal. We implemented feature selection algorithms to enhance the IDS performance in the IoT devices. Therefore, since our goal is to construct an effective IDS that can detect attacks on Internet of Things devices within the IoT network dataset, we examined a variety of machine learning algorithms to choose which models were the most accurate and efficient learner. Using the scaling of the data and the selection of features, we construct and evaluate four supervised models. RF, DT, LR, and KNN are the models that are included in the proposed model. In addition, to improve the effectiveness of our method for attack detection, we employ two ensemble methods to improve the efficiency of the proposed model. The performance evaluation includes accuracy, precision, recall, and F1-score metrics.

Furthermore, the main objective is to use a variety of supervised machine learning models to combine and input them into the ensemble model, which offers a solution for enhancing the IDS performance besides predicting malicious and normal IoT network traffic, where 0 denotes normal, and 1 means abnormal, using the features type, date, ts, time, label, longitude, altitude, and label. However, in the result section, we will illustrate the final result for all ML classifiers.

**Figure 1:**
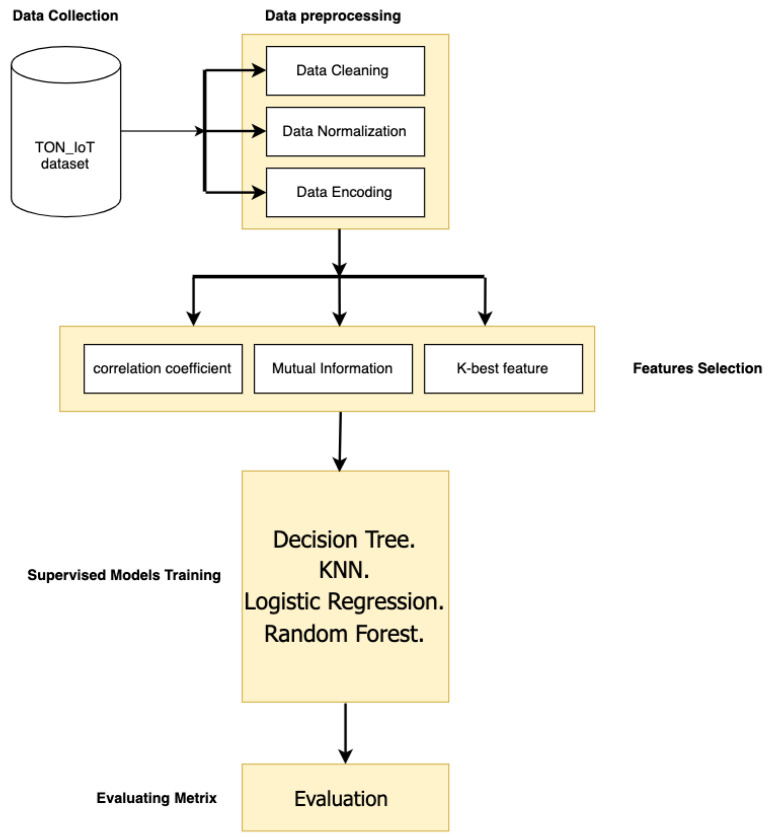
Illustration the workflow of the supervised ML classifiers that are applied to IDS.

Moreover, Figure 1 illustrates the first workflow of the supervised ML classifiers applied to the IDS model. While it presents the preprocessing process and the methods used to fulfill all required steps to complete the preprocessing requirement, the proposed model used data cleaning, label encoding, and data normalization processes. In addition, Figure 1 illustrates the feature selection methods applied to the proposed model: Mutual information (MI), Pearson Coefficient Correlation (PCC), and K-Best feature. After we selected the proposed model features based on the feature selection methods, we trained the model to conduct the results. Finally, we will discuss the ML models, the preprocessing process, and feature selection methods applied to the proposed model.

#### 4.1.1. Random forest (RF)

RF is described as a Random decision forest and a Machine learning algorithm that can be applied to several methods, such as ensemble learning. The samples for the forest decision [42] were generated by the RF, which is structured by constructing an unconnected tree and then centralizing it. Although RF is applied to deal with high-dimensional data, a significant difficulty with RF is that it has to cope with vast or huge data while being a viable technique to enhance accuracy by applying and merging numerous decision trees DTs [28]. Although it utilized memory and demanded additional time since it depends on a computational process, it gave high-accuracy results because it deals with dimensional data to improve performance.

#### 4.1.2. Decision Tree (DT)

DT is a well-known Machine learning ML classification used to build an Intrusion detection system. DT contains three essential factors: the decision node, the branch, and leaf node. After the model was learned and trained with the dataset, the DT established and formed the decision tree [42]. DT is characterized by the fact that it can handle numerical and categorical features and recognize nonlinear relationships. Despite the simplicity of implementation of the DT, DT requires and consumes more storage capacity, which is considered an issue [59]. In our experiment, the DT presented high-accuracy results with straightforward implementation while requiring more space in memory. The mathematical representation of functionality is as follows:

(1) $$G\left(D\right)=\Sigma_{I=1}^{C}(P\left(i\right)*\left(1-P\left(i\right)\right)$$

The Gini impurity is a measure of the level of impurity or uncertainty in a training dataset (*D*) that is constructed from a set of class labels (*C*) and the fractions (*p*(*i*)) of samples that are assigned to each class label (*I*) in the set. The Gini impurity is zero when there is just one class label in the set.

#### 4.1.3. K-Nearest Neighbors (KNN)

KNN refers to K-Nearest Neighbors, where K is the number of the selected sample points. KNN is a supervised machine learning algorithm applied to a set of data points or a single one to make a classification or prediction. While KNN deals with regressions and classification issues, the KNN works based on the hypothesis that a point can be located near another with similarity [60]. Furthermore, the voting majority generates the class point, which appears most frequently in specified data points. When KNN is defined as a suitable method to handle multi-classification tasks, KNN has an issue: the values of K will be different from one dataset to another [60]. While KNN has influenced our model positively since it deals with complex decisions borderline, it consumes memory and requires more time since it relies on computational processes.

Moreover, KNN uses the Euclidean distance to calculate the spread between the nearest points to measure the KNN represented in the equation as [60]:

(2) $$Euclideandistance=\sqrt{\left({\left(a1-a2\right)}^{2}+\left(b1-b2\right)\right)}$$

Which, (*a*1 − *a*2) is the first, and (*b*1 − *b*2) is the second.

#### 4.1.4. Logistic Regression (LR)

The LR algorithm can be used to classify a set of discrete variables. The logistic sigmoid is the basis for logistic regression (LR). This technique predicts the probability value of a test sample that can be plotted to discrete types of two or more by transforming absolute values into values between 0 and 1 [42]. The corresponding transactions are classified as negative when the model predicts a less than 50 result. When the outcome is anticipated to be greater than 50, the corresponding transactions are classified as positive. is the equivalent mathematical expression [42]. The mathematical representation of functionality is as follows:

(3) $$g\left(z\right)=\frac{\left(1\right)}{\left(1+e^{-2}\right)}$$

An estimated probability between 0 and 1 is represented by the function *g*(*z*), where *z* is the input value, *e* is the base of the logarithm of nature, and *z* is the result.

### 4.2. Ensemble Learning (EL)

**Figure 2:**
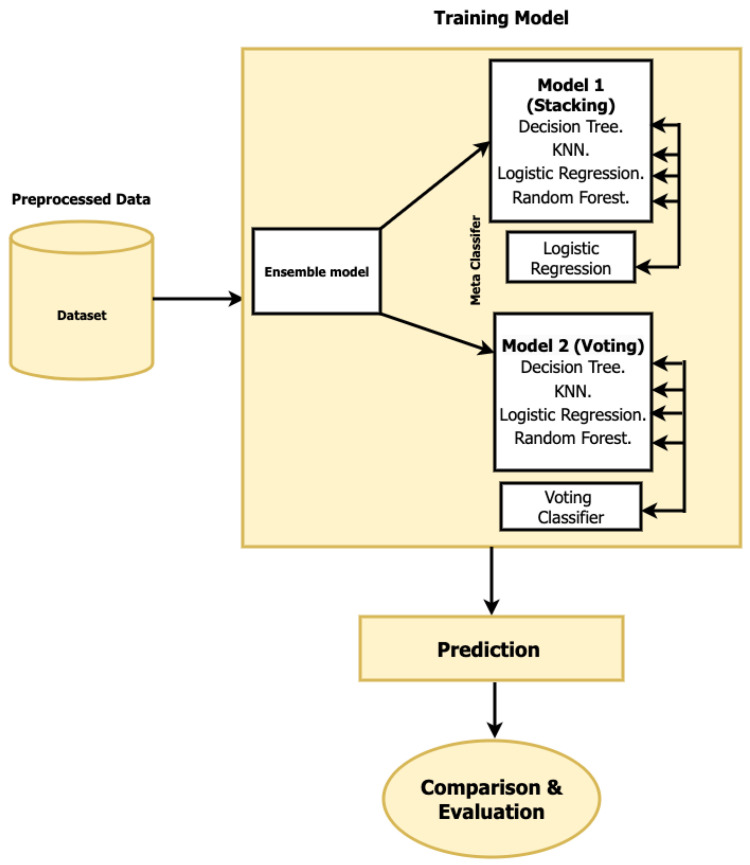
Illustration the workflow of the proposed ensemble classifiers.

Instead of applying learning techniques and algorithms one at a time, EL allows you to view them simultaneously by combining a group of classifiers or predictors. These groups of classifiers are trained and learned from prior patterns of behavior to address complex issues [17,61]. The workflow of the proposed ensemble classifier approach is described in Figure 2 for any application.

The supervised model used the preprocessed dataset, as shown in Figure 1 in Section 4. While Figure 2 indicates that the stacking and voting ensemble classifiers were examined, it represents the second workflow of the proposed model. We used ensemble classifier prediction approaches to improve our system’s accuracy. We aimed to enhance classification accuracy by merging many classifiers into a single ensemble platform. We will give each classifier’s findings and explain and compare the two approaches in the results section. Figure 2 illustrates the workflow of the proposed ensemble classifiers.

Furthermore, We build and assess four supervised models using data scaling and the feature selection. This group of models consists of Random Forests, Decision Trees, Logistic Regression, and K-Nearest Neighbors to improve IDS detection capability. We employ two ensemble methods to enhance the attack detection efficiency of our scheme. This system uses ensemble classifiers based on classification algorithms. We chose stacking and voting as ensemble techniques since their predictions are weighted depending on the relevance of the individual classifiers. The weighted probabilities are then added together to get the total probability.

### 4.3. TON-IoT Network Dataset

The TON IoT network traffic dataset is acquired from real-world IoT network device (in-home) scenarios [18,19,20,21,22,23,24,25]. The TON-IoT dataset network was generated from UNDW Canberra Cyber, the Australian Defense Force Academy (ADFA), housed in the School of Engineering and Information Technology (SEIT) at the University of New South Wales (UNSW) in Canberra [20]. The TON-IoT datasets, collected from multiple IoT devices through IoT architecture, have been tested and authenticated in a cyber lab to be more reliable to be applied and implemented through machine learning techniques.

In this study, however, we present a binary classification of normal and abnormal IoT device network traffic. The proposed model presents a solution for improving IDS performance by constructing an efficient ensemble framework of supervised machine learning models.

Moreover, several datasets have been used for IDS domain development, such as BOT IOT (2018), UNSWNB (2015), and KDD-CUP99, the most common datasets. Still, these datasets have shown that a lack of various sensors and newly updated attacks may lead to a need for more data. Consequently, TON-IoT was selected for several IDS approaches, while it is publicly available and the most recent work to build an IDS. The TON means the telemetry data, system log operating, and network traffic of the IoT network from which the dataset was collected. Thus, the dataset has multiple dataset files containing sensor telemetry data, so Table 4 presents details of the selected files in the proposed model.

In the proposed model, we have combined all the datasets mentioned above into one dataset called the combined dataset to establish the experiment and presented model. In contrast, the TON-IoT dataset contains telemetry data in all dataset files representing various IoT devices and realistic IoT network traffic with different attack scenarios. In addition, Table 5 Presents statistical details of the attack types in the combined dataset.

### 4.4. Data Preprocessing

#### 4.4.1. Data Cleaning

Several individual dataset files exist within the TON-IoT dataset network; we merged them into one single document called the combined dataset. Even though the new, larger file resulted from the merge, we still had to clean up some unexpected information, so random data as ‘-’ has been removed from several features. Therefore, the median value process was utilized to recalculate values for each feature in place of the extracted and missing values. The median was chosen as the measurement method instead of the mean because it is more robust against outlier errors [32].

#### 4.4.2. Label Encoding

In addition, many ML classifiers work exclusively with continuous values; hence, features with categorical values must be transformed into continuous ones. Since the combined dataset has several categorical features required to be transformed into numerical values, we employed the Label Encoding approach. Label Encoding was chosen because it does not increase the number of features or the computational complexity of the modeling process. Both are important considerations given the time needed for classification [32,42]. The natural values of true, false, off, and low were consequently converted to zero or one.

#### 4.4.3. Data Standardization and Normalization

Moreover, most classification algorithms function more effectively when features are of comparable magnitude, since this helps to lessen the bias toward traits with high multiplicity values in the prediction results [32]. Although the combined dataset has a variety of attribute values, the fact that several features contain values in a wide range (from zero to hundreds to thousands) would negatively impact the model’s performance and produce erroneous results. Therefore, standardization and normalization for scaling the features are two methods for dealing with this problem. As a result, we used min-max scaling for several features in our investigation and provided a model. The following equation represents the Min-Max scaling method [32]:

(4) $$a_{normlized}=\frac{{(}^{a-a}min)}{{(}^{a}max{-}^{-a}min)}$$

The dataset maintains attribute ‘a’ values. ‘amax’ and ‘amin’ indicate attribute ‘a’’s greatest and lowest values. Data separation preceded feature scaling. To preserve the prediction accuracy of trained models, the normalized data in the training set was kept hidden from the test data [32].

#### 4.4.4. Data Splitting

It is necessary to note that the data has been partitioned per the feature mapping. The dataset was split into training and testing subsets using stratification and randomization, with the same 70:30% split of class types as the original dataset. Predictive models were developed using the training data, and the best intrusion detection model was selected based on its performance in the testing set.

### 4.5. Features Selections

Additionally, experiments are run to evaluate various feature selection algorithms and determine which ones are most effective for detecting anomalies. The training process can be simplified through feature selection by eliminating irrelevant or redundant data points from the collection [32]. Mutual information, Pearson Coefficient Correlation, and K-Best are the most popular and common feature selection methods that are applied for various IDS models:

#### 4.5.1. Mutual Information (MI)

Is the one that has been modified the most frequently in practice. A reduction in uncertainty for one variable is predicted by this formula when the other variable has a known value. The value of mutual information indicates a stronger relationship between the two variables if it is greater than zero [32]. A result of 0 from a calculation including both variables suggests that they are unrelated. Thus, the mathematical representation of the MI is as follows [32]:

(5) IG(X|Y)=H(X)−H(X|Y)

where H(x) is the the process of the independent variable x, given by

(6) $$H\left(X\right)=-\Sigma_{i}P\left(x_{i}\right)log_{2}\left(P\left(x_{i}\right)\right)$$

as well as the formula for H(x|y):

(7) $$H\left(x|y\right)=-\Sigma_{i}P\left(y_{i}\right)\Sigma_{i}P\left(x_{i}|y_{i}\right)log_{2}\left(P\left(x_{i}|y_{i}\right)\right)$$

#### 4.5.2. Pearson Coefficient Correlation (PCC)

Represents a standard to evaluate the degree of a statistical association between two independent variables [32]. Its value, which can range from −1 to 1, and its sign, which can be negative or positive, indicate the type of significance between two attribute vectors. This formula is used to determine PCC [32]:

(8) $$r=\frac{\Sigma \left(x-x^{-}\right)\left(y-y^{-}\right)}{\sqrt{\Sigma {\left(x-x^{-}\right)}^{2}+\Sigma {\left(y-y^{-}\right)}^{2}}}$$

The symbol r denotes Pearson’s correlation coefficient (PCC). The variable x represents the conditional feature, and the value y represents the decision feature. In addition, we refer to the sample means for the x and y characteristics as x and y, respectively.

#### 4.5.3. K-Best Feature

Each feature’s value is computed, then the chi-squared function is applied to those values, and finally, the top feature’s value is ranked according to the k we choose. The chi-squared statistic [62] is used in feature selection to determine whether or not two features are independent of a given class. This equation is written mathematically as [32]:

(9) $$x^{2}\left(f,c\right)=\frac{m*{\left(PQ-RT\right)}^{2}}{\left(P+x)+(P+Q)+(P+R)+(T+Q\right)}$$

where f stands for a single feature, c for a class, M for the entire dataset’s total number of occurrences, P is the frequency, f occurs in c, and Q is the frequency neither f nor c does. The frequency with which class c occurs without feature f, denoted by R, and class f without class c, represented by T, are inversely proportional.

Furthermore, feature selection uses chi-squared statistics. This statistic determines if two class-related features are independent. The chi-squared test determines a feature’s importance to the target class. This equation’s numerical address can determine the feature’s importance. Thus, the highest-scoring features are modeled. Chi-squared feature selection improves model efficiency in several ways: Removing unneeded or duplicate features reduces dimensionality during feature selection. This reduces overfitting and improves model performance. Selecting the most relevant attributes helps researchers determine which data features best predict the target class. Readability may help uncover data patterns and correlations. Removing unnecessary characteristics might help the model focus on data values, improving generalization. This enhances the model’s pattern recognition and prediction. Chi-squared-based feature selection helps us select the most relevant characteristics, minimize dimensionality, improve interpretability, and increase model generalization [63].

After we applied the above selection method to the combined dataset, we decided to select the most repeated features given high scores by the selection algorithms, and we called the selected models features (Final selection). Table 6 illustrates the features that each method has selected.

## 5. Results

This section compares the learning models and the experimental results with traditional performance metrics like F1-score, recall, accuracy, and precision. The models created after the ML bais models algorithms have been trained and evaluated based on standard key evaluation parameters [64]:

1. True positive (TP): The method correctly identified and categorized malicious attempts across many samples [58].
2. True Negative (TN): The algorithm accurately categorizes the proportion of normal samples [58].
3. True positive (TP): The method correctly identified and categorized malicious attempts across many samples [64].
4. True Negative (TN): The algorithm accurately categorizes the proportion of normal samples [64].

The performance measures generated from the parameters above are described as follows:

### 5.1. Accuracy

The accuracy metric is necessary to determine how well the model is doing. On the other hand, it is only usable with data that is evenly distributed [58,64]. It is computed as the ratio of correctly predicted occurrences to the total test samples. The mathematical representation of the equation is as follows [64]:

(10) $$Accuracy=\frac{TP+TN}{TP+TN+FP+FN}$$

### 5.2. Precision

The percentage of accurate predictions that turned out to be accurate [58]. In other words, the proportion of positively identified samples that were correctly classified (TP) compared with the balance of positively identified samples that were mistakenly classified (but were still positively identified) [64]. The mathematical representation of the equation is as follows:

(11) $$Precision=\frac{TP}{FP+TP}$$

### 5.3. Recall

The percentage of the positive sample count that has been correctly categorized and identified [64]. The equation is as follows:

(12) $$Recall=\frac{TP}{FN+TP}$$

### 5.4. F1-Score

The F1-score, with its usual range of 1.0 to 0.0, can be used to determine the harmonic mean of precision and recall [64]. The F-1 score improves as the degree of accuracy and precision increases. The equation is as follows [58]:

(13) $$F1-\mathrm{score}=2*\frac{Precision*Recall}{Precision+Recall}$$

While we got our data from the TON-IoT dataset, as stated, we started the prepatory process to build the supervised machine learning models. In addition, four effective classifiers were employed to detect normal and abnormal IoT network traffic. LR, k-NN, RF, and DT were used. The primary goal is to combine them into the ensemble to enhance the IDS performance.

Moreover, Table 7 illustrates the evaluation results of the supervised models. LR has achieved the highest accuracy, 98.42%; the KNN has 98.28% accuracy; the RF has 98.15% accuracy; and the DT has 97.44% accuracy, which is considered the lower one, respectively.

Our research aims to develop a binary classification based on an ensemble-based learning model to boost the IDS’s efficiency further. The ensemble-based learning approach combined four supervised ML models (LR, KNN, DT, and RF). These models are accurate and well-balanced in terms of data, while LR, KNN, and RF stand out as having the highest accuracy. The advantages of the ensemble learning approach become apparent when considering how different learning strategies can mutually benefit one another. Stacking and voting are two methods of ensemble classifier models. Table 8 highlights the ensemble classifiers and the performance metrics for the two ensemble classifier models.

We chose stacking and voting as ensemble techniques since their predictions are weighted depending on the relevance of the individual classifiers. The weighted probabilities are then added together to get the total probability. Furthermore, stacking involves two steps. There are four model-based learners (LR, KNN, DT, and RF) in the first step; the next stage is to include one model in a meta-learner (Logistic Regression). Stacking uses these two stages to learn and identify the best approach to merging base and meta-learner models. Stacking outperformed Voting in all measures metrics.

**Figure 3:**
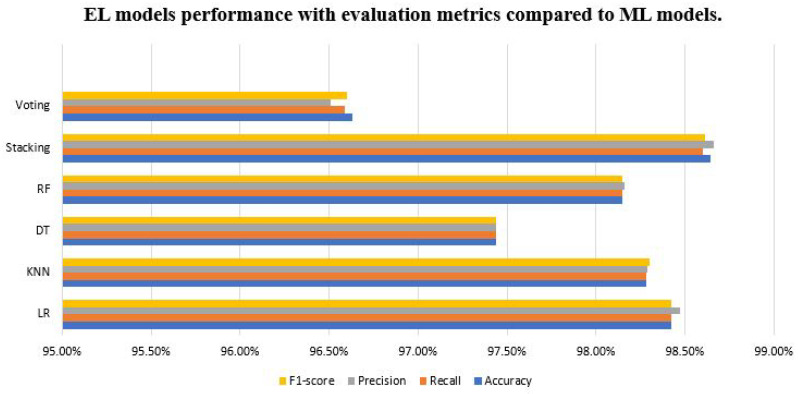
Compares the proposed and supervised ML models regarding evaluation metrics.

However, Figure 3 illustrates the comparison process between the ensemble methods and the supervised ML models, which emphasizes that the proposed stacking model outperforms the Voting ensemble classifier, although the supervised ML models have achieved high accuracy. Stacking has the highest F1 scores (98.64%), Accuracy (98.64%), Recall (98.60%), and precision (98.20%).

Table 3 presents recent machine-based learning models that used ensemble approaches in refs. [17,28,42] and were compared with our proposed scheme. Both of them used the same dataset. The proposed model performed better when measured against evaluation metrics for the same dataset. While the proposed IDS can detect attacks on IoT network traffic, the detection performance is improved based on the conducted results. The suggested ensemble models for binary classification showed performance and evaluation metrics improvements compared with the prior models.

### 5.5. Discussion

Table 3 compared the performance metrics of different approaches on the TON-IoT and CICIDS2017 datasets. Each approach utilizes different machine learning algorithms.

Moreover, the research [53] used the TON-IoT dataset, the Random Forest (RF), XGBoost (XGBoos), LightGBM (LGBM), and CatBoost algorithms were employed. The approach achieved recall, precision, F1-score, and accuracy in the range of 91% to 97%, 89% to 97%, 89% to 97%, and 89% to 97%, respectively. In the study [42] on the same TON-IoT dataset, the Logistic Regression (LR), Linear Discriminant Analysis (LDA), RF, Naive Bayes (NB), Support Vector Machine (SVM), and Long Short-Term Memory (LSTM) algorithms were utilized. The reported metrics for this approach were 85% recall, 87% precision, 86% F1-score, and 86% accuracy. In addition, the study [28] employed the CICIDS2017 dataset and used the Naive Bayes (NB), Decision Tree (DT), and Logistic Regression (LR) algorithms. Although specific values are provided for recall, precision, and F1-score, the reported accuracy was 88.92%.

The proposed models on the TON-IoT dataset employed the Random Forest (RF), K-Nearest Neighbors (KNN), Logistic Regression (LR), and Decision tree (DT) algorithms. The stacking and voting methods used in our model to generate these results. The evaluation metrics for our ensemble stacking model were 98.60% recall, 98.20% precision, 98.61% F1-score, and 98.63% accuracy. Our models are compared with recently proposed models that utilized the same dataset and similar approaches. Our models performed better at detecting attacks and presented an enhancement in the IDS performance, as shown in Table 6, Table 7 and Table 8. Precision is 98.2, recall is 98.60, the F1 score is 98.61, and accuracy is 98.63.

Moreover, stacking and voting were selected as ensemble methods because their predictions are weighted according to the importance of the individual classifiers. After assigning weights, probabilities can be totaled to generate an overall prediction. The proposed stacking model performs better than the proposed ensemble classifier and is highly accurate, although supervised ML models are also highly accurate. F1 (98.64%), Accuracy (98.60%), Recall (98.2%), and Precision (98.1%) are all best achieved by the stacking method. There are two phases of the Stack. In the first phase, we employ four model-based learners (Logistic Regression, K-Nearest Neighbor, Distance-based, and Radial Basis Function). These two steps allow stacking to learn and determine the optimal method for combining base and meta-learner models. Compared with Voting, the Stacking method performed better on all measures and metrics.

These evaluation metrics have shown improvement over prior research. The proposed model uses RF, KNN, LR, and DT algorithms. Combining multiple algorithms into an “ensemble”, the model uses their strengths to generate better outcomes than a single algorithm. The proposed model stacks many distinct models and integrates their predictions with a meta-model. The model can detect patterns and make more accurate predictions by combining basic model results.

Furthermore, we relied on the ensemble since the ensemble learning approach allows all learning techniques and algorithms to be evaluated simultaneously rather than individually. There has been an increase in the usage of EL for prediction and forecasting in recent years, and this has allowed it to be put to use in solving a number of challenging tasks. EL relies on a set of combined classifiers or predictors instead of single classifiers, so these sets of classifiers are trained and learned from the conducted patterns to address the same issue and get better results.

While we proposed a binary classification of IoT device network traffic as normal or abnormal, by combining the four supervised ML models, we employed two ensemble methods to improve the efficiency of the proposed model. The proposed method primarily focuses on the Internet of Things (IoT); however, it is noteworthy that the fundamental techniques and approaches are transferable to other network environments. The principles of supervised machine learning, ensemble classifiers, and enhancing detection efficacy are not exclusive to the Internet of Things (IoT) and can be customized to various network contexts.

Hence, the proposed approach is tailored to optimize Intrusion Detection System (IDS) efficacy in the Internet of Things (IoT). However, this approach’s fundamental principles and methodologies can potentially be extrapolated and implemented in other network contexts. While we evaluated the proposed model in the IoT network traffic dataset, the proposed methodology may provide valuable perspectives and methods that have the potential to enhance intrusion detection across diverse network environments, extending beyond the boundaries of the Internet of Things.

## 6. Conclusions

This study proposes an IDS for IoT networks that employs machine learning to identify malicious behavior in IoT network data. The primary objective of this approach is to enhance the efficiency of intrusion and attack detection. This research compares and contrasts four supervised machine learning algorithms—Random Forest, Decision Tree, Logistic Regression, and K-Nearest Neighbor— to classify normal and abnormal Internet of Things network data. Logistic Regression and K-Nearest Neighbor classifiers provided the most optimal findings. Ensemble approaches, including voting and stacking, are used to improve classification by combining all supervised models. By working together, various ensemble learning methods can improve classification accuracy.

Ensemble learning has the advantage of combining various learning methods to support and reinforce one another in a classification task. This means that the efficiency of the machine learning models is greatly improved by the ensemble classifiers, which perform better than the individual supervised classifiers. Performance measures highlight stacking over voting, with improved accuracy, recall, and F1-Score. In particular, the stacking model performs exceptionally well in terms of recall. The stacking method has an F1 score of 0.986 and scores of 0.9864 for accuracy, 0.9864 for precision, 0.9864 for recall, and 0.9864 for both. Compared with the previously used models, these enhancements constitute a major advance forward. Moreover, as shown in Table 8, stacking shows improvement in accuracy, precision, and F1-score compared with the models referenced in [28,42,53]. Our objective for the future is to develop an innovative multi-classification approach that can detect and classify anomalies and intrusions in IoT network traffic.

## Figures and Tables

**Table 1:** State-of-the-Art Learning Based approach.

Paper	Dataset	Potential Approach	Classification Type	Attacks
[8]	BoT-IoT	Learning model (RF, and CNN)	Binary	Botent IoT malware, and IoT network attacks traffic.
[52]	Distilled-Kitsune-2018	Ensemble-based learning	Binary	OS scan, ARP, SSDP, SSL, and Maria attacks.
[53]	KDD cup’99 and NSL-KDD	Naïve Base, KNN, RF, and DT algorithms.	Binary	DoS, Sybil and Spoofing, Man-in-the-middle, Hole ataacks.
[54]	NSL-KDD	Ensemble-based learning	Multi-Class	DDoS Attacks, and IoT network attacks traffic.
[55]	Network Traffic	Ensemble-based learning	Binary/Multi-Class	Data theft, DoS, and spam
[56]	NSL-KDD	Ensemble-based learning	Binary	DDoS Attacks, and IoT network attacks traffic.
[57]	IoT Network Traffic	K-means	Binary	Ping flood Attacks

**Table 2:** Recent Machine based learning applying Ensemble methods proposed model.

Paper	Year	Dataset	Objectives	Accuracy	Approach
**[45]**	2022	TON_IoT	Comparison process of ensemble learning for multiclass IoT network attacks.	91–96%	RF, XGBoos, LGBM, and CatBoost
**[46]**	2022	TON_IoT	Ensemble learning with binary and multi-class classification to enhance IDS system performance.	86%	LR, LDA, RF, NB, SVM, and LSTM
**[47]**	2022	CICIDS2017	A Binary ensemble-based learning for improving the performance of IDS system	88%	NB(M), DT, and LR
**The proposed Model**	2023	TON_IoT	A Binary ensemble-based learning for improving the performance of IDS system with a Comparison process of ensemble learning	98%	RF, ET, KNN, and SVC, Stacking method

**Table 3:** Recent Ensemble-learning based methods compared with the proposed approach.

Paper	Year	Dataset	Recall	Precision	F1-Score	Accuracy	Approach
[53]	2022	TONIoT	91–97%	89–97%	89–97%	89–97%	RF, XGBoos, LGBM, and CatBoost
[42]	2022	TONIoT	85%	87%	86%	86%	LR, LDA, RF, NB, SVM, and LSTM
[28]	2022	CICIDS2017	Nil	Nil	Nil	88.92%	NB(M), DT, and LR
The proposed Model	2023	TONIoT	98.60%	98.20%	98.61%	98.63%	RF, ET, KNN, and SVC Stacking method

**Table 4:** Details the selected files in the proposed model.

Datasets	Number of Features	Number of Instances	Features’ Names	Types of Attacks
TON_IoT (IoT_Fridge).	7	59,944	Ts, date, time, fridge_temperature, temp_condition, type, and label.	
TON_IoT (IoT_Garage_Door).	15	409,963	ts, date, time, door_state, sph1e_signal, temp_c1diti1, l1gitude, light_status, current_temperature, thermostat_status, temperature, pressure, humidity, type, and lable	
TON_IoT (IoT_Thermostat).	7	52,774	Ts, date, time, current_temperature, thermostat_status, type and label.	
TON_IoT (IoT_GPS_Tracker).	7	58,960	Ts, date, time, latitude, longitude, type and label.	
TON_IoT (IoT_Motion_Light).	7	59,488	Ts, date, time, motion_status, light_status, type and label.	
TON_IoT (IoT_Weather).	8	59,260	Ts, date, time, temperature, pressure, humidity, type and label.	
Combined_dataset	20	700,389	ts, date, time, fridge_temperature, temp_condition, label, type, door_state, sph1e_signal, temp_c1diti1, l1gitude, light_status, current_temperature, thermostat_status, temperature, pressure, humidity, latitude, longitude, moti1_status	Normal. Backdoor. DDoS. Injection. Password Ransomware. Scanning. XSS.

**Table 5:** Presents statically details of the attacks types [42].

Data No.	Attack Types							Attacks Totals	Normal Totals
	Scanning	DDOS	Ransomware	Backdoor	Injection	XSS	Password		
1	Nil	5000	2902	5000	5000	2042	5000	24,944	35,000
2	529	5000	2902	5000	5000	1156	5000	24,587	35,000
3	61	Nil	2264	5000	5000	449	5000	17,774	35,000
4	550	5000	2833	5000	5000	577	5000	23960	35,000
5	1775	5000	2264	5000	5000	449	5000	24,488	35,000
6	529	5000	2865	5000	5000	866	5000	24,260	35,000
7	3444	25,000	13,990	30,000	30,000	5539	30,000	185,494	190,710

1. TON-IoT (IoT-Fridge). 2. TON-IoT (IoT-Garage-Door). 3. TON-IoT (IoT-Thermostat). 4. TON-IoT (IoT-GPS-Tracker). 5. TON-IoT (IoT-Motion-Light). 6. TON-IoT (IoT-Weather). 7. Combined data.

**Table 6:** Illustrates the features that each method has selected and the final selection.

Feature Selection Technique	Selected Features
**Mutual information (MI)**	type, date, ts, time, and lable
**Pearson Coefficient Correlation (PCC)**	date, l1gitude, light_status, longitude, moti1_status, sph1e_signal, temp_condition, and type, lable.
**K-Best feature**	type, date, ts, time, logtiude, and iltitude.
**Final selection**	type, date, ts, time, lable, logtiude, iltitude nad lable

**Table 7:** Evaluation results of the Supervised models.

Algorithm	Accuracy	Recall	Precision	F1-Score
**LR**	98.42%	98.42%	98.47%	98.42%
**KNN**	98.28%	98.28%	98.29%	98.30%
**DT**	97.44%	97.44%	97.44%	97.44%
**RF**	98.15%	98.15%	98.16%	98.15%

**Table 8:** Evaluation results of the ensemble classifiers.

Algorithm	Accuracy	Recall	Precision	F1-Score
**Stacking**	98.64%	98.60%	98.66%	98.61%
**Voting**	96.63%	96.59%	96.51%	96.60%

## Data Availability

Data available upon request.
